# Fucoidan improving spinal cord injury recovery: Modulating microenvironment and promoting remyelination

**DOI:** 10.1111/cns.14903

**Published:** 2024-08-14

**Authors:** Haoming Shu, Xin Zhang, Yingyan Pu, Yinuo Zhang, Shixue Huang, Jun Ma, Li Cao, Xuhui Zhou

**Affiliations:** ^1^ Department of Orthopedics, Second Affiliated Hospital Naval Medical University Shanghai China; ^2^ Department of Neurobiology, Key Laboratory of Molecular Neurobiology of the Ministry of Education Naval Medical University Shanghai China; ^3^ Department of Orthopedics, Shanghai General Hospital Shanghai Jiao Tong University School of Medicine Shanghai China

**Keywords:** fucoidan, microenvironment, neuroinflammation, OPCs differentiation, remyelination, spinal cord injury

## Abstract

**Introduction:**

Excessive neuroinflammation, apoptosis, glial scar, and demyelination triggered by spinal cord injury (SCI) are major obstacles to SCI repair. Fucoidan, a natural marine plant extract, possesses broad‐spectrum anti‐inflammatory and immunomodulatory effects and is regarded as a potential therapeutic for various diseases, including neurological disorders. However, its role in SCI has not been investigated.

**Methods:**

In this study, we established an SCI model in mice and intervened in injury repair by daily intraperitoneal injections of different doses of fucoidan (10 and 20 mg/kg). Concurrently, primary oligodendrocyte precursor cells (OPCs) were treated in vitro to validate the differentiation‐promoting effect of fucoidan on OPCs. Basso Mouse Scale (BMS), Louisville Swim Scale (LSS), and Rotarod test were carried out to measure the functional recovery. Immunofluorescence staining, and transmission electron microscopy (TEM) were performed to assess the neuroinflammation, apoptosis, glial scar, and remyelination. Western blot analysis was conducted to clarify the underlying mechanism of remyelination.

**Results:**

Our results indicate that in the SCI model, fucoidan exhibits significant anti‐inflammatory effects and promotes the transformation of pro‐inflammatory M1‐type microglia/macrophages into anti‐inflammatory M2‐type ones. Fucoidan enhances the survival of neurons and axons in the injury area and improves remyelination. Additionally, fucoidan promotes OPCs differentiation into mature oligodendrocytes by activating the PI3K/AKT/mTOR pathway.

**Conclusion:**

Fucoidan improves SCI repair by modulating the microenvironment and promoting remyelination.

## Graphical abstract

Our results indicate that in the SCI model, fucoidan exhibits significant anti‐inflammatory effects and promotes the transformation of pro‐inflammatory M1‐type microglia/macrophages into anti‐inflammatory M2‐type ones. Fucoidan enhances the survival of neurons and axons in the injury area and improves remyelination. Additionally, fucoidan promotes OPC differentiation into mature oligodendrocytes by activating the PI3K/AKT/mTOR pathway. These findings suggest that fucoidan improves SCI repair by modulating the microenvironment and promoting remyelination.

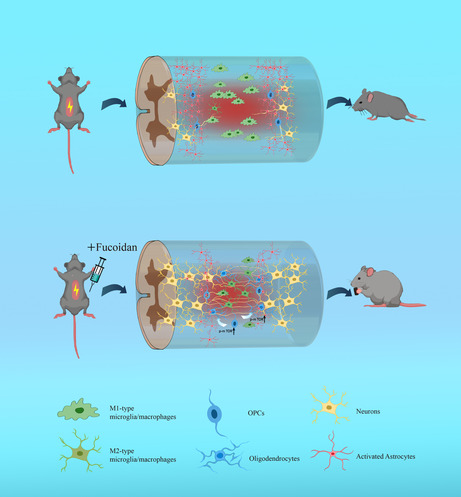

## 1 INTRODUCTION

Spinal cord injury (SCI) is devastating damage to the spinal cord, leading to serious dysfunction of sensation and locomotion and even death. The consequences that come with SCI include physical and mental damage, decreased quality of life, and a huge economic burden on society and patients with SCI. The SCI pathophysiological process is complex and considered to be initiated by the primary injury, followed by the secondary injury.^1^ The primary injury is the mechanical force, commonly in the form of contusion or compression, acting on the spinal cord, resulting in blood–spinal cord barrier (BSCB) damage, nerve cell membrane disruption, and axon rupture. Secondary injury is the characteristic phase of SCI involving neuronal apoptosis, oxidative stress, demyelination, inflammation, and glial scar formation.^2^, ^3^, ^4^ Extensive studies focusing on the damage factors have been performed but have shown limited therapeutic efficacy.^5^

Following SCI, innate immune cells, including microglia, astrocytes, and endothelial cells, respond rapidly and release cytokines and chemokines. Classical inflammatory cytokines, such as interleukin‐1 beta (IL‐1β), interleukin‐6 (IL‐6), and tumor necrosis factor‐alpha (TNF‐α), are upregulated within hours of SCI, leading to the assemblage of microglia and macrophages in the lesion core.^6^, ^7^ Furthermore, these assembled immune cells secrete additional pro‐inflammatory cytokines that result in exacerbation of inflammation. The cascade‐amplified inflammatory response produces many oxygen free radicals, destroys cells, triggers necrosis and apoptosis of nerve cells, exacerbates the formation of glial scars, and destroys the BSCB.^8^ The aforementioned factors ultimately contribute to the formation of an inhibitory injury microenvironment and hinder the process of SCI recovery.^9^ Additionally, demyelination, which commences shortly after SCI, has a detrimental impact on neural signal transmission and can cause axon disruption.^10^ Therefore, promoting the survival, repair, and regeneration of the myelin sheath is crucial in the treatment of SCI.

Currently, therapeutic options for SCI are severely limited in scope. Methylprednisolone is widely used in clinical practice, but its dosage is positively correlated with the risk of complications such as gastrointestinal bleeding, hyperglycemia, and infection.^11^ Aiming at developing effective and safe treatment methods for SCI, we screened for novel drugs that exhibit neuroprotective effects and promote myelin regeneration. Natural medicines have attracted our attention due to their low side effects. Fucoidan (Fuc) is a natural plant metabolite with highly attractive biological activities, such as anti‐inflammation, antioxidation, immunomodulation, and neuroprotection.^12^, ^13^, ^14^

Fuc has been confirmed to exert a beneficial therapeutic role in several central nervous system diseases, including Alzheimer's disease, Parkinson's disease, stroke, and brain injury.^15^, ^16^ In studies related to Alzheimer's disease, Fuc showed anti‐aggregation effects against amyloid fibrils and exert neuroprotection effects against Aβ‐induced neurotoxicity.^17^ In a rotenone‐induced rat model of PD, chronic treatment with fucoidan significantly reversed the loss of nigral dopaminergic neurons by enhancing mitochondrial respiratory function.^15^ Particularly, recent findings have indicated that Fuc could effectively protect neurons from transient global cerebral ischemia through attenuation of activated glial cells and reduction of oxidative stress by increasing superoxide dismutase (SOD) activity.^18^ It has also been reported that Fuc has a significant protective effect on the progression of traumatic brain injury in mice.^19^ However, it was still unclear whether fucoidan had beneficial effects in treating SCI.

In the present study, using a SCI mice model, we demonstrated that the administration of Fuc could suppress early inflammatory responses and, consequently, improve functional recovery following SCI and especially promote remyelination in the lesion region. In vitro experiments further demonstrated that this regenerative effect on myelin is associated with the role of Fuc in promoting OPCs differentiation and maturation through the PI3K/AKT/mTOR signaling pathway. The findings shed light on re‐purposing Fuc to treat SCI.

## 2 MATERIALS AND METHODS

### 2.1 Cells and cell culture

The isolation and purification of mouse primary OPCs/microglia were conducted according to established protocols with modifications tailored to our experimental setup. Briefly, cortical tissues from postnatal day 0 (P0) mice were dissected, homogenized, and cultured in poly‐D‐lysine‐coated T75 culture flasks containing DMEM/F12 supplemented with 10% fetal bovine serum (FBS) at 37°C in an atmosphere containing 5% CO_2_. The cultures were maintained for 10–12 days with regular medium changes every 3 days. The culture flasks were subjected to agitation at 180 rpm for 1 h and the medium was collected for primary microglia culture. The flasks was filled with fresh medium and shaken at 200 rpm for 18 h. Subsequently, the conditioned medium was collected and allowed to adhere to uncoated dishes for 40 min, promoting the attachment of microglia and astrocytes while preventing OPCs attachment. The resulting OPCs‐enriched suspension was then reseeded onto poly‐D‐lysine‐coated plates or coverslips at densities ranging from 5000 to 50,000 cells/cm^2^. For differentiation assays, OPCs were cultured in neurobasal medium supplemented with 2% B27 for 3 days prior to experimental analysis.

Primary cortical neurons were isolated from the cerebral cortex of P0 mice. Briefly, after the removal of blood vessels and meninges, the brain tissues were finely chopped into approximately 1 mm^3^ fragments and digested with 0.25% trypsin–EDTA and DNase I (MilliporeSigma) for 30 min. The digestion was terminated using 10% FBS, followed by centrifugation at 1000 rpm for 6 min. The cells were then seeded onto poly‐D‐lysine‐coated plates and incubated in neurobasal medium supplemented with 2% B27 and 1% penicillin/streptomycin. The culture medium was refreshed by replacing half of it every 2 days.

### 2.2 Animals

Male C57/BL6 mice aged 8–9 weeks were procured from Shanghai Jihui Laboratory Animal Co., Ltd. (Shanghai, China) for all animal experiments. Mice were housed under standardized conditions and maintained on a 12‐h light–dark cycle. All animal experiments were conducted following approval from the Animal Welfare and Research Ethics Committee of Shanghai Changzheng Hospital (Shanghai, China) and in strict accordance with the guidelines of the Chinese National Institutes of Health.

### 2.3 SCI model

A crushed SCI model was performed as previously reported.^20^ The animals were randomly assigned to one of three groups: vehicle, Fuc (10 mg/kg), and Fuc (20 mg/kg), with 20 animals per group. A schematic diagram illustrating the experimental setup is provided in Figure 2A. Anesthesia was induced using 3% inhaled isoflurane (Yaji Biological Technology, Shanghai, China) and maintained with 1.5% isoflurane. Subsequently, a laminectomy was performed at the T7‐9 level to expose the spinal cord. Spinal cord trauma was induced by laterally compressing the T8 spinal cord using a pair of forceps with a 0.40 mm spacer for 15 s.

### 2.4 Drug treatment

**FIGURE 1:**
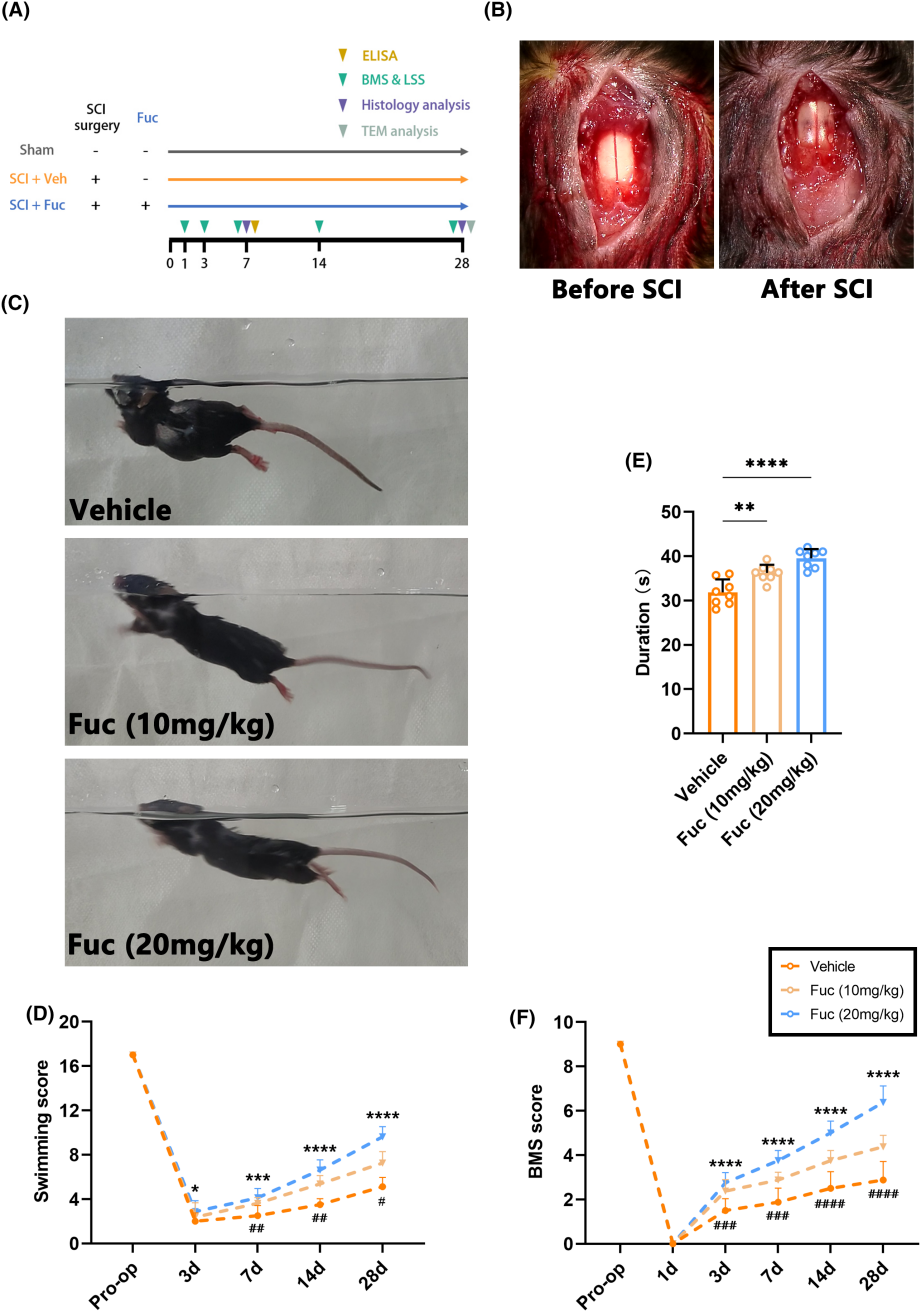
Fucoidan administration promotes locomotor recovery. (A) Schematic diagram of SCI modeling and fucoidan treatment. (B) Dorsal view of mouse spinal cord before and after injury. (C) Representative images of swimming test in different groups. (D) The LSS of SCI mice treated with saline, fucoidan(10 mg/kg), fucoidan(20 mg/kg) during the recovery period 28 days after injury. (two‐way ANOVA). (E) The Rotarod test revealed the latency in the time to fall from the spinning drum at 28 dpi. (one‐way ANOVA). (F) The BMS scores of SCI mice treated with saline, fucoidan (10 mg/kg), fucoidan (20 mg/kg) during the recovery period 28 days after injury. (two‐way ANOVA). The data in D–F passed the Shapiro–Wilk test and exhibit a Gaussian distribution. Data are presented as mean ± SEM, (*n* = 8 mice). For D, F ^#^Vehicle versus Fuc (10 mg/kg), *Vehicle versus Fuc (20 mg/kg). ^##^ *p* < 0.01, ^###^ *p* < 0.001, ^####^ *p* < 0.0001, *p < 0.05, **p < 0.01, ***p < 0.001, ^****^ *p* < 0.0001, ^#^ *p* < 0.05.

Fuc (HY‐132179, MedChemExpress) was dissolved in normal saline for in vivo experiments and PBS for in vitro experiments. Mice in both Fuc groups received intraperitoneal (i.p) injections of Fuc at dosages of 10 and 20 mg/kg, respectively, as indicated in Figure 1A, while animals in the vehicle grou*p we*re administered an equivalent volume of normal saline.

### 2.5 Immunofluorescence staining

For tissue samples, three mice per group were euthanized and transcardially perfused with PBS, followed by 4% paraformaldehyde solution at 8 weeks post‐injury. Spinal cord samples from the T11–12 region were harvested and post‐fixed in 4% paraformaldehyde solution for 12 h. Subsequently, the samples were dehydrated and embedded in OCT (Sakura) before being sectioned into 10 μm sections using a cryo‐ultramicrotome. Antigen retrieval was performed on sections using citrate buffer (0.01 M, pH 6).

For cell samples, cells were fixed in 4% paraformaldehyde for 30 min, followed by blocking with PBST blocking buffer containing 10% normal donkey serum. Primary antibodies, including anti‐MBP (1:200, MAB386, MilliporeSigma), anti‐caspase 3 (1:500, 9664, Cell Signaling Technology), anti‐NF200 (1:2000, ab207176, Abcam), anti‐CC1 (1:100, MABC200, MilliporeSigma), anti‐SOX10 (1:200, AF2864, R&D Systems), anti‐GFAP (1:500, ab7260, Abcam), anti‐NeuN (1:1000, ABN90, MilliporeSigma), anti‐Arg1 (1:50016001‐1‐AP, Proteintech), anti‐iNOS (1:500, 18985‐1‐AP, Proteintech), and anti‐CD68 (1:500, ab31630, Abcam) were incubated at 4°C overnight, followed by secondary antibodies (1:200, Jackson ImmunoResearch) at room temperature for 2 h. The sections were visualized using an Olympus IX70 microscope system (Olympus, Tokyo). Six randomly selected non‐overlapping fields from each section were analyzed for quantification.

### 2.6 BrdU incorporation

To evaluate the proliferation of OPCs, 5‐bromo‐2‐deoxyuridine (BrdU, 10 μM; Sigma) was added to the culture medium 6 h prior to fixation in 4% paraformaldehyde solution. Subsequently, coverslips were incubated in 2 N HCl for 30 min, followed by treatment with 0.1 M borate buffer (pH 8.0) for 25 min to denature DNA. Permeabilization of OPCs was achieved using 0.2% Triton X‐100 for 2 min, followed by blocking with PBST containing 10% donkey serum for 1 h at room temperature. Coverslips were then incubated overnight with anti‐BrdU antibody (1:100, B35128, Invitrogen). After washing with PBS, cells were incubated with secondary antibodies (1:200, Jackson ImmunoResearch) and DAPI (Sigma) for 1 h at room temperature.

### 2.7 TUNEL staining

In this experiment, tissue samples consisted of slices of spinal cord tissue harvested on day 7 post‐injury, while cell samples comprised OPCs subjected to a 3‐day differentiation period. Apoptotic cells were identified and quantified using a TUNEL Assay Kit (Beyotime), following the manufacturer's instructions. Additionally, the nuclei of all cells were counterstained with DAPI. Three sections were randomly selected from each group, and within each section, three fields of view were randomly chosen. The number of positive cells within these fields was then counted with ImageJ software (NIH, Bethesda, MD, USA).

### 2.8 Live/dead staining

After 7 days of in vitro culture, the medium of primary cortical neurons was replaced with fresh medium containing hydrogen peroxide (H_2_O_2_, 100 μM, MilliporeSigma) for an additional 24 h. The cells were then randomly divided into four groups: a sham group (no H_2_O_2_ treatment), a vehicle control group (H_2_O_2_ treatment), and two Fuc groups (treated with H_2_O_2_ and Fuc at two concentrations).

Following the instructions provided in the live/dead staining kit (Solarbio Science & Technology), the culture medium was removed, and the cells were washed twice with 1× Assay Buffer for 2 min each. After adding Calcein‐AM staining solution, the cells were incubated at 37°C in the dark for 20 min. PI staining solution was then added, and the cells were stained in the dark at room temperature for 5 min. The staining solution was removed, and the cells were washed twice with 1× PBS for 2 min each. Finally, the slides were mounted and observed under a fluorescence microscope.

### 2.9 Western blot analysis

Tissue and cell protein extraction reagents were used for total protein sample preparation. The protein concentration was determined using the BCA Protein Assay Kit (Beyotime) according to the manufacturer's instructions. Equivalent quantities of protein samples were loaded and separated on sodium dodecyl sulfate polyacrylamide‐gel electrophoresis (SDS‐PAGE) at 140 V for approximately 90 min. Protein transfer from polyacrylamide gels to nitrocellulose (NC) membranes (Merck) was achieved by placing them together in an electrophoresis bath. Following protein transfer, membranes were blocked in 10% (w/v) milk at room temperature for 1 h, followed by overnight incubation with primary antibodies, including anti‐ARG1 (1:1000, 16001‐1‐AP, Proteintech), anti‐iNOS (1:1000, K0221, Santa Cruz), anti‐MBP (1:2000, AB7349, Abcam), anti‐p‐mTOR (1:1000, 5536P, Cell Signaling Technology), and anti‐mTOR (1:1000, 2893P, Cell Signaling Technology), anti‐PI3 Kinase (1:500, 9242, Cell Signaling Technology), and anti‐Phospho‐PI3 Kinase p85 (1:500, 19366S, Cell Signaling Technology) at 4°C. After washing with TBST, the membranes were incubated with corresponding HRP‐conjugated secondary antibodies (1:10,000, Proteintech) at room temperature for 1 h. Protein bands were visualized using Chemiluminescent HRP Substrate (WBKLS0500, Millipore), and Image Lab 4.1 software (Bio‐Rad) was used for protein band quantification.

### 2.10 Enzyme‐linked immunosorbent assay (ELISA)

TNF‐α, IL‐10 in the supernatant of microglia were measured by ELISA kit (Multi Sciences) following the manufacturer's instructions.

### 2.11 Transmission electron microscopy (TEM)

For TEM examination, spinal cord specimens underwent a specialized preparation procedure. Mice were euthanized with isoflurane and transcardially perfused with PBS and 4% paraformaldehyde solution. Subsequently, the spinal cord columns were isolated and consecutively fixed in 2.5% glutaraldehyde for 2 h, followed by 1% OsO_4_ for 45 min. Following fixation, samples were dehydrated using a gradient of ethanol (30%–100%) and embedded in Araldite resin. Sections (1 μm) were stained with toluidine blue, while ultrathin sections (60 nm) were cut using copper grids and stained with uranyl acetate (3%) and lead citrate (1%). TEM was performed using a transmission electron microscope (H‐7650, Hitachi) at an accelerating voltage of 100 kV.

### 2.12 Basso Mouse Scale (BMS)

Hindlimb motor function was assessed using the BMS prior to surgery and on postoperative days 1, 3, 7, 14, and 28. The BMS scoring system assigns a score ranging from 0 to 9, where a score of 0 denotes no ankle movement and a score of 9 represents complete functional recovery. Two independent researchers conducted the observation and recording of hindlimb movements of animals.

### 2.13 Louisville Swim Scale (LSS)

Hindlimb function, trunk instability, and body angle were assessed using the LSS prior to injury and at 3, 7, 14, and 28 days post‐SCI. The LSS assigns a score ranging from 0 to 17, where 0 indicates no hindlimb movement, and 17 indicates normal hindlimb movement. Each mouse was pre‐trained to swim. Two independent researchers recorded the average score of each mouse.

### 2.14 Rotarod test

A rotarod apparatus equipped with an acceleration setting (ranging from 0 to 40 rpm) was used to evaluate gross motor function and coordination in mice. Each mouse underwent one practice trial, followed by two test trials, with a 20 min interval between trials. The average latency to fall from the rotarod was calculated based on the results of the two test trials for each animal.

### 2.15 Statistical analysis

All data analyses were performed using GraphPad Prism 9.0. For normality and lognormality tests, a Shapiro–Wilk was performed. For the comparison between groups, statistical significance was determined using the unpaired two‐tailed Student's *t*‐test. For multiple comparisons, a one‐way ANOVA with Fisher LSD test or Dunnett's multiple comparisons test for pairwise comparisons in multiple groups (for variables with homogenous variance) or Games–Howell (for variables with non‐homogenous variance), or two‐way ANOVA, followed by Tukey's multiple comparisons, was performed. Data are presented as the mean ± S.E.M. for at least three independent biological replicates. The “*n*” numbers for each experiment are specified in the figure legends. The three‐dimensional surface plot was generated with the ImageJ 1.52v. A value of *p* < 0.05 was considered statistically significant.

## 3 RESULTS

### 3.1 Fuc promotes locomotor function after SCI

To ascertain whether Fuc confers protective effects against SCI, we conducted animal experiments, as illustrated in Figure 1A, and assessed the motor function of mice. In short, Fuc is administered by i.p injection from day 1 to day 28 after SCI. Mice in the sham group only underwent spinal cord exposure without subsequent steps of injury; they were considered to be free of behavioral and histochemical staining defects and were only present as a positive reference group in TEM. In addition to histochemistry and TEM on day 7 and day 28 after SCI, both the vehicle group and the Fuc treatment groups underwent behavioral tests on the dates indicated in the experimental procedure.

The motor behavior of SCI mice was initially assessed using the BMS score. As shown in Figure 1F, all mice that underwent injury exhibited complete hindlimb paralysis (BMS score = 0) on day 1 after SCI, indicating successful and homogeneous modeling. Beginning at 3 days post injury(dpi), the animals demonstrated gradual, time‐dependent recovery, with varying degrees of improvement observed in all groups. Compared to the vehicle group, mice treated with Fuc demonstrated a trend toward higher BMS score elevation at 3–28 dpi, suggesting that Fuc treatment significantly promotes locomotor recovery. The results of the experiment showed BMS scores were significantly higher in the SCI + Fuc (10 mg, 20 mg/kg) group compared to the vehicle group (7, 14, and 28 dpi).

Swimming exactly reflects the motor capacity of the animal's nervous system. Hence, we performed swimming tests at multiple time points after SCI and used the LSS for quantitative assessment^21^ (Figure 1C,D). As shown in Figure 1D, the motor function of all groups gradually recovered from day 7 after SCI, and the recovery rate was significantly faster in the Fuc‐treated groups, especially in the 20 mg/kg group.

In the rotarod test performed on day 28 after SCI, the Fuc‐treated groups demonstrated better balance and motor coordination on all tests compared to the vehicle group (Figure 1E). Taken together, these results indicated that Fuc treatment notably improved the motor function of SCI mice.

### 3.2 Fuc reduces early inflammatory response after SCI and regulates microglial/macrophages polarization

**FIGURE 2:**
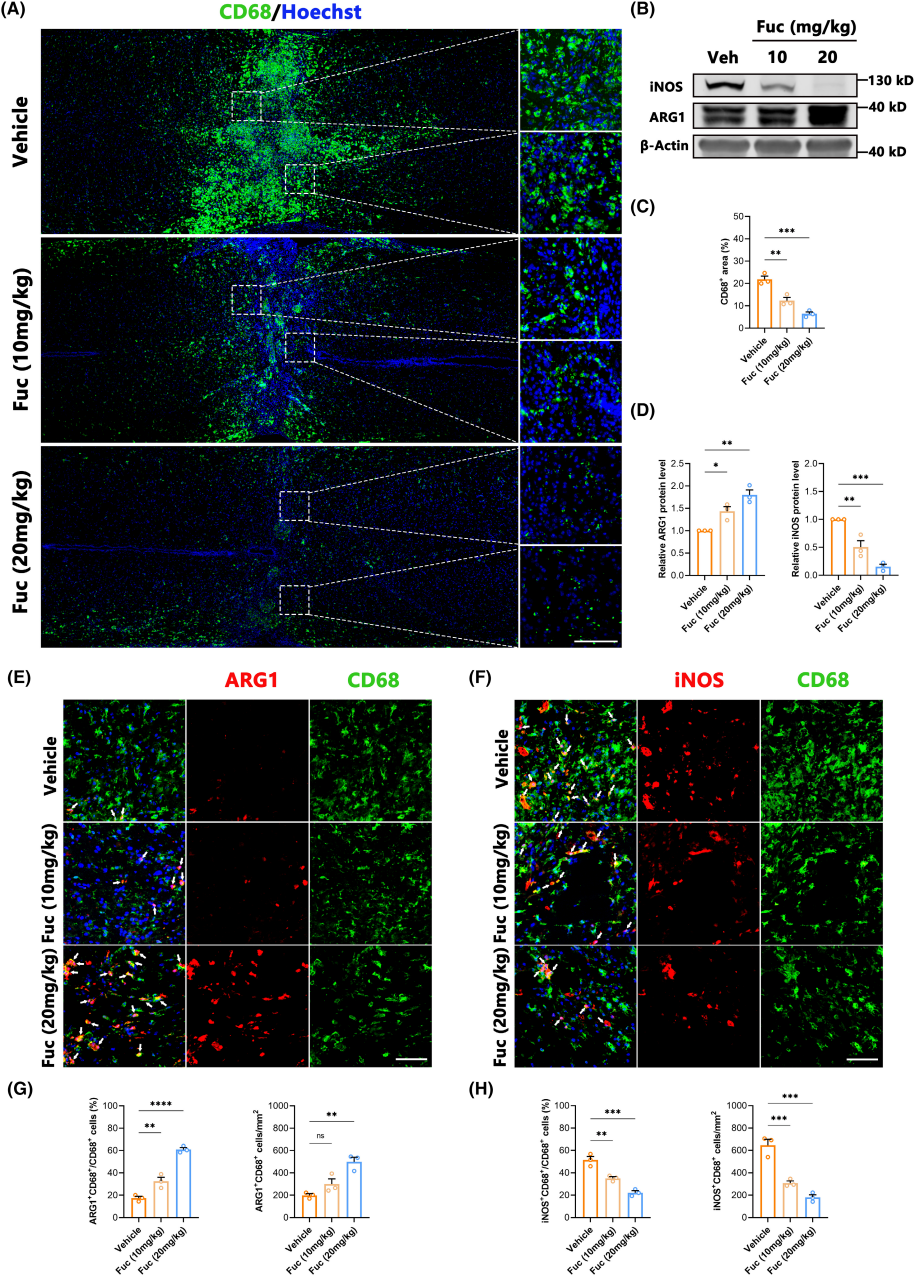
Fucoidan attenuates neuroinflammation in vivo by promoting microglia anti‐inflammatory polarization and inhibiting pro‐inflammatory polarization after SCI. (A) Immunofluorescence staining of CD68 (green) from SCI mice with different treatments at 7 dpi. Nuclei were counterstained with Hoechst (blue). Magnifications of the boxed regions are shown on the right. (scale bar, 100 μm). Vehicle, SCI group. Fuc (10 mg/kg), SCI treated with 10 mg/kg Fucoidan group. Fuc (20 mg/kg), SCI treated with 20 mg/kg Fucoidan group. (B) Western blotting analysis of iNOS and ARG1 expression in spinal cord obtained at 7 dpi. (C) Quantification analysis of the number of CD68^+^ cells obtained from three regions of interest (ROIs) at 7 dpi (one‐way ANOVA). (D) Quantification of the relative expression levels of ARG1 protein and iNOS protein as in B (one‐way ANOVA). (E) Immunofluorescence co‐staining of CD68 (green), ARG1 (red) from SCI mice with different treatments at 7 dpi. Nuclei were counterstained with Hoechst (blue). White arrows mark ARG1^+^CD68^+^ cells. (scale bar, 100 μm). Vehicle, SCI group. Fuc (10 mg/kg), SCI treated with 10 mg/kg Fucoidan group. Fuc (20 mg/kg), SCI treated with 20 mg/kg Fucoidan group. (F) Immunofluorescence co‐staining of CD68 (green), iNOS (red) from SCI mice with different treatments at 7 dpi. Nuclei were counterstained with Hoechst (blue). White arrows mark iNOS^+^CD68^+^ cells. (scale bar, 100 μm). Vehicle, SCI group. Fuc (10 mg/kg), SCI treated with 10 mg/kg Fucoidan group. Fuc (20 mg/kg), SCI treated with 20 mg/kg Fucoidan group. (G) The ratio of ARG1^+^CD68^+^/CD68^+^ cells and the quantitative analysis of the number of ARG1^+^CD68^+^ cells obtained from three ROIs at 7 dpi (one‐way ANOVA). (H) The ratio of iNOS^+^CD68^+^/CD68^+^ cells and the quantitative analysis of the number of iNOS^+^CD68^+^ cells obtained from three ROIs at 7 dpi (one‐way ANOVA). The data in C, D, G, and H passed the Shapiro–Wilk test and exhibit a Gaussian distribution. Data are presented as mean ± SEM, (*n* = 3 mice), **p < 0.01, ***p < 0.001, ^****^ *p* < 0.0001, ns, not significant.

Inflammation represents a pivotal target for SCI treatment, as its regulation can mitigate a spectrum of secondary damages and improve the injury microenvironment.^22^, ^23^ In our study, we used Iba1 as the marker to characterize microglia and macrophages, employing iNOS as a marker for M1‐type inflammatory cells and Arg1 as a marker for M2‐type ones.^24^ Our findings (Figure 2A,C) distinctly illustrate that Fuc intervention significantly restricted the inflammatory extent within the injury site, concomitant with a marked reduction in inflammatory cell numbers. Correspondingly, the levels of iNOS and ARG1 proteins assessed by Western blotting were significantly higher in SCI lesions. Fuc treatment decreased the expression of iNOS while concurrently increasing that of ARG1 (Figure 2B,D). Furthermore, Fuc not only curbed inflammation but also reversed the polarization of inflammatory cells (Figure 2E–H). We also detected the levels of pro‐inflammatory cytokine (TNF‐α) and anti‐inflammatory cytokine (IL‐10) in the supernatant of microglia stimulated by LPS for 1 day using ELISA. After Fuc treatment, especially high‐dose Fuc treatment, the level of TNF‐α significantly decreased while the level of IL‐10 significantly increased (Figure S2). As aforementioned, this suggests that Fuc not only confines inflammation spread and diminishes injury scope but also transitions inflammatory cells from a pro‐inflammatory to an anti‐inflammatory, pro‐repair state.

### 3.3 Fuc promotes neuronal survival and suppresses apoptosis after SCI

**FIGURE 3:**
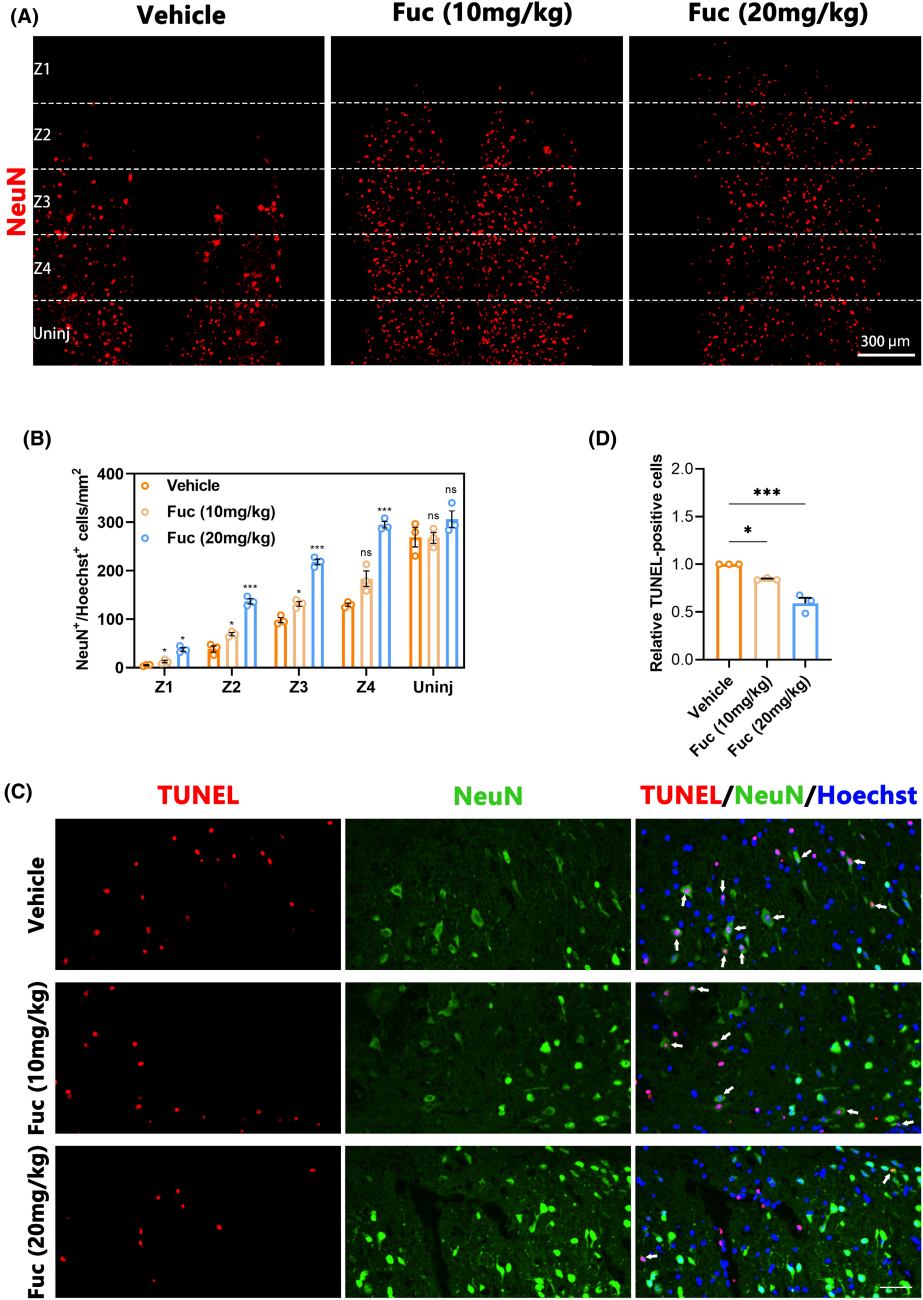
Fucoidan facilitates neuronal retention by reducing apoptosis in vivo. (A) Representative immunofluorescence images of NeuN^+^ cells (red) in the Z1–Z4 region adjacent to the lesion core at 28 dpi. Uninj, uninjured. (scale bar, 300 μm). Vehicle, SCI group. Fuc (10 mg/kg), SCI treated with 10 mg/kg Fucoidan group. Fuc (20 mg/kg), SCI treated with 20 mg/kg Fucoidan group. (B) Quantification of NeuN^+^ neurons in A. (two‐way ANOVA). (C) Representative images showing TUNEL^+^(red) NeuN^+^ (green) cells in different treatment groups at 7 dpi. Nuclei were counterstained with Hoechst (blue). White arrows mark TUNEL^+^ NeuN^+^ cells. (scale bar, 100 μm). Vehicle, SCI group. Fuc (10 mg/kg), SCI treated with 10 mg/kg Fucoidan group. Fuc (20 mg/kg), SCI treated with 20 mg/kg Fucoidan group. (D) Quantitative analysis of the relative percentages of TUNEL^+^ cells obtained from three ROIs at 7 dpi (one‐way ANOVA). The data in B, D passed the Shapiro–Wilk test and exhibit a Gaussian distribution. Data are presented as mean ± SEM, (*n* = 3 mice), *p < 0.05, ***p < 0.001, ns, not significant.

Following SCI, the survival of neurons at the injury site directly influences motor and sensory functions in mice.^25^ To understand the anatomical basis of functional recovery in the treatment group, we conducted NeuN staining to identify surviving neurons and quantified the number of NeuN^+^ cells at various distances from the injury center in coronal slices across different experimental groups. In the Z1–Z3 region, the number of surviving neurons in the Fuc‐treated groups was significantly higher than in the vehicle group, with the neuroprotective effect of the Fuc 20 mg/kg group being superior to that of the Fuc 10 mg/kg group (Figure 3A,B). To further investigate the benefits of Fuc in SCI repair, primary cortical neurons were treated with H_2_O_2_ (100 μM) for 24 h to mimic neuronal injury. In the in vitro neuronal injury model, Fuc significantly enhanced neuronal survival, with higher concentrations exhibiting greater protective effects (Figure S1A–C).

Apoptosis is a critical mechanism of neuronal cell death following SCI,^26^ and its severity directly impacts neuronal survival. We performed TUNEL staining on mice sampled 7 days post‐SCI to assess apoptosis levels. As illustrated in Figure 3C, the number of TUNEL‐positive cells in the Fuc treatment group was significantly lower than in the vehicle group. Furthermore, compared to the Fuc 10 mg/kg group, the Fuc 20 mg/kg group exhibited a stronger anti‐apoptotic effect (Figure 3C,D). These TUNEL findings corroborate the neuronal staining results. In all, these results collectively confirm the neuroprotective role of Fuc in inhibiting neuronal apoptosis post‐SCI and promoting neuronal survival. Additionally, this protective effect was more pronounced in the Fuc 20 mg/kg group than in the Fuc 10 mg/kg group.

### 3.4 Fuc alleviates glial scar formation and promotes axonal regeneration following SCI

**FIGURE 4:**
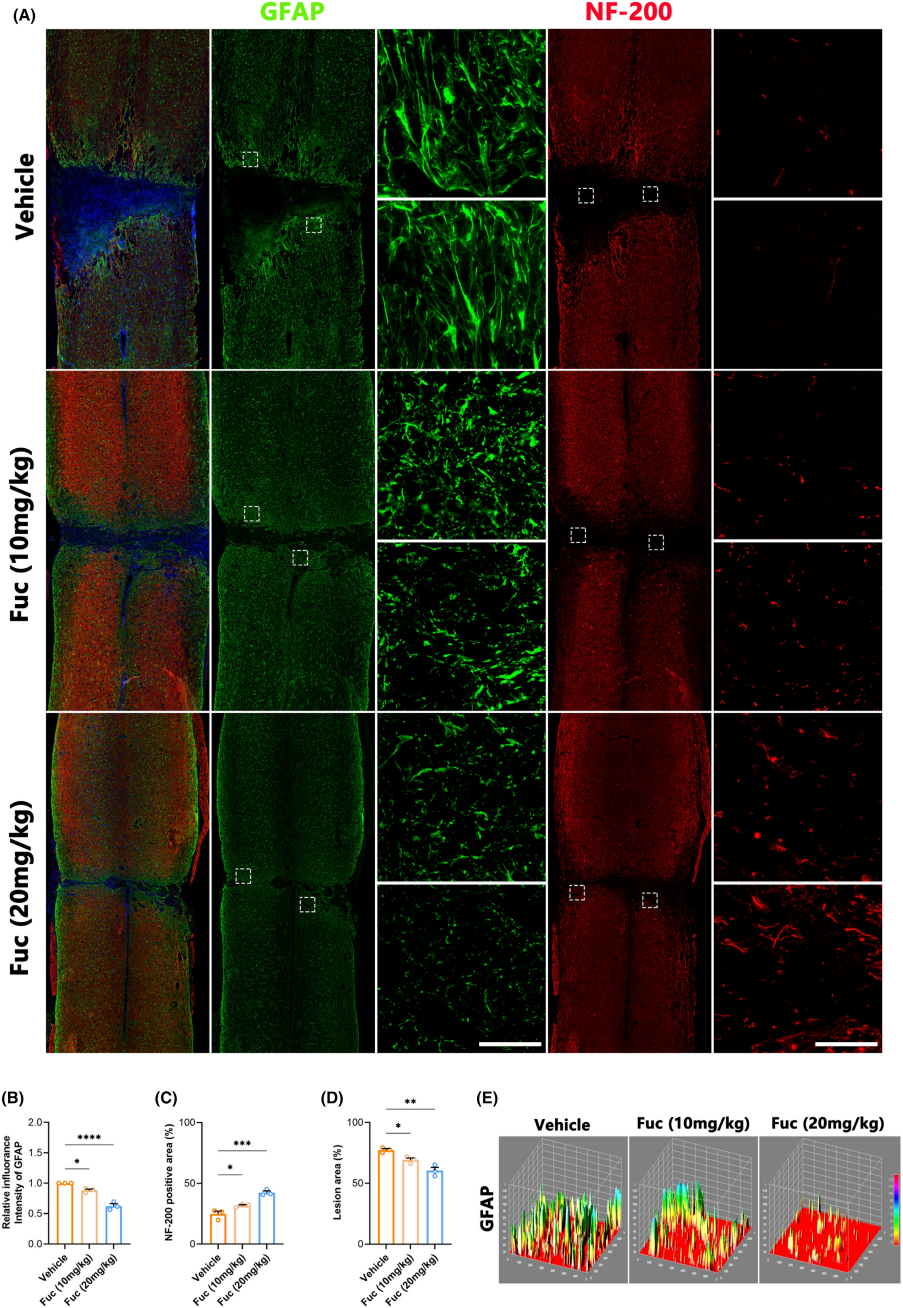
Fucoidan promotes axonal regeneration and inhibits glial scar formation after SCI. (A) Immunofluorescence co‐staining of NF‐200(red), GFAP(green) from SCI mice with different treatments at 28 dpi. Magnifications of the boxed regions are shown on the right. (scale bar, 60 μm). Vehicle, SCI group. Fuc (10 mg/kg), SCI treated with 10 mg/kg Fucoidan group. Fuc (20 mg/kg), SCI treated with 20 mg/kg Fucoidan group. (B) Quantification of the fluorescence intensity of GFAP obtained from three ROIs at 28 dpi (one‐way ANOVA). (C) Quantification of the percentage of NF200^+^ area obtained from three ROIs at 28 dpi (one‐way ANOVA). (D) Quantification of the percentage of lesion area obtained from three ROIs at 28 dpi (one‐way ANOVA). (E) Representative of the 3D surface plot of GFAP in the lesion region of each group (Red represents low value, green represents high value). The data in B–D passed the Shapiro–Wilk test and exhibit a Gaussian distribution. Data are presented as mean ± SEM, (*n* = 3 mice), *p < 0.05, **p < 0.01, ***p < 0.001, ^****^ *p* < 0.0001.

The primary manifestation of neurological dysfunction following SCI is the disruption of neural pathways, with axons serving as fundamental conduits for nerve impulse transmission.^27^ Therefore, assessing axonal repair is crucial in evaluating SCI treatments. While astrocytic scars are thought to limit inflammation and protect nerve cells in the early stages of SCI, they subsequently impede axonal regeneration and extension, posing a significant obstacle to spinal cord repair.^28^ To investigate neural repair post‐SCI in our study, we used GFAP as an astrocyte activation marker and NF200 to characterize neural axons. As depicted in Figure 4A–E, astrocytes proliferate and accumulate at the injury edge, forming a glial scar area post‐injury. Compared to the vehicle group, scar density significantly decreased in both treatment groups, with the high‐dose group (Fuc 20 mg/kg) demonstrating the most pronounced effect. Consistent with this finding, there was a significant increase in the number of axons within the injury area in the treatment groups, particularly in the Fuc 20 mg/kg group.

### 3.5 Fuc promotes maturation of OPCs in SCI lesions

**FIGURE 5:**
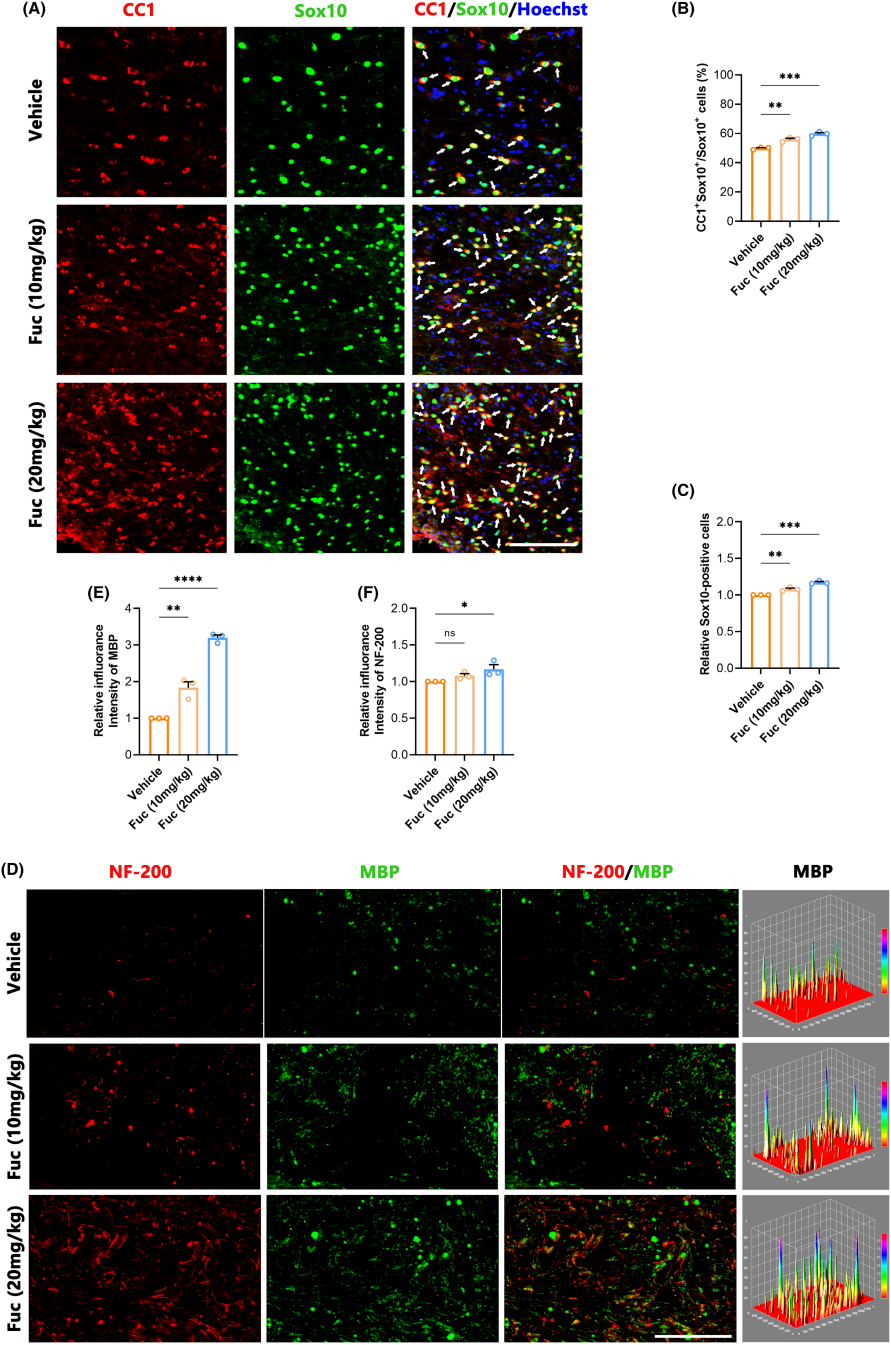
Fucoidan promotes axonal regeneration and myelination. (A) Immunofluorescence co‐staining of CC1 (red), Sox10 (green) from SCI mice with different treatments at 28 dpi. Nuclei were counterstained with Hoechst (blue). White arrows mark CC1^+^ SOX10^+^ cells. (scale bar, 100 μm). Vehicle, SCI group. Fuc (10 mg/kg), SCI treated with 10 mg/kg Fucoidan group. Fuc (20 mg/kg), SCI treated with 20 mg/kg Fucoidan group. (B) The ratio of CC1^+^Sox10^+^/ Sox10^+^ cells obtained from three ROIs at 28 dpi (one‐way ANOVA). (C) Quantitative analysis of the relative percentages of Sox10^+^ cells obtained from three ROIs at 28 dpi (one‐way ANOVA). (D) Immunofluorescence co‐staining of MBP (red), NF‐200 (green), and the representative of the 3D surface plot of MBP from SCI mice with different treatments at 28 dpi. (scale bar, 100 μm). Vehicle, SCI group. Fuc (10 mg/kg), SCI treated with 10 mg/kg Fucoidan group. Fuc (20 mg/kg), SCI treated with 20 mg/kg Fucoidan group. (E) Quantification of the fluorescence intensity of MBP in D (one‐way ANOVA). (F) Quantification of the fluorescence intensity of NF‐200 in D (one‐way ANOVA). The data in B, C, E, and F passed the Shapiro–Wilk test and exhibit a Gaussian distribution. Data are presented as mean ± SEM, (*n* = 3 mice), *p < 0.05, **p < 0.01, ***p < 0.001, ^****^ *p* < 0.0001, ns, not significant.

Following the attenuation of the acute inflammatory response, SCI transitions to its chronic phase, marked by myelin regeneration, vascular remodeling, and neural circuit reorganization.^29^ In this investigation, we performed immunostaining on spinal cord sections, employing CC1 to identify mature oligodendrocytes^30^ and SOX10 to label cells within the OL lineage.^31^ Double immunofluorescence staining in Figure 5A showed that among the groups, more SOX10^+^ cells appeared in the lesion region of the Fuc treatment groups, especially in the 20 mg/kg group (Figure 5C). Fuc (20 mg/kg) treatment also increased the proportion of CC1^+^ cells among SOX10^+^ cells (Figure 5B). These results collectively indicate that Fuc not only enhances the number of OL‐lineage cells but also promotes the maturation of OPCs.

### 3.6 Fuc promotes myelination after SCI

To evaluate the regeneration of myelin in the lesion region, we used NF‐200 to label axons^32^ and MBP to label myelin sheaths.^33^ Due to the observation of more axons and stronger OPC differentiation in the Fuc treatment groups than in the vehicle group, we further examined myelination in the injury area. As shown in the immunofluorescence double staining for MBP and NF200 (Figure 5E), there were more MBP^+^ and NF200^+^ signals in the Fuc treatment groups (Figure 5F,G), indicating an enhancement in myelination along with the increase in axon numbers compared to the vehicle group.

**FIGURE 6:**
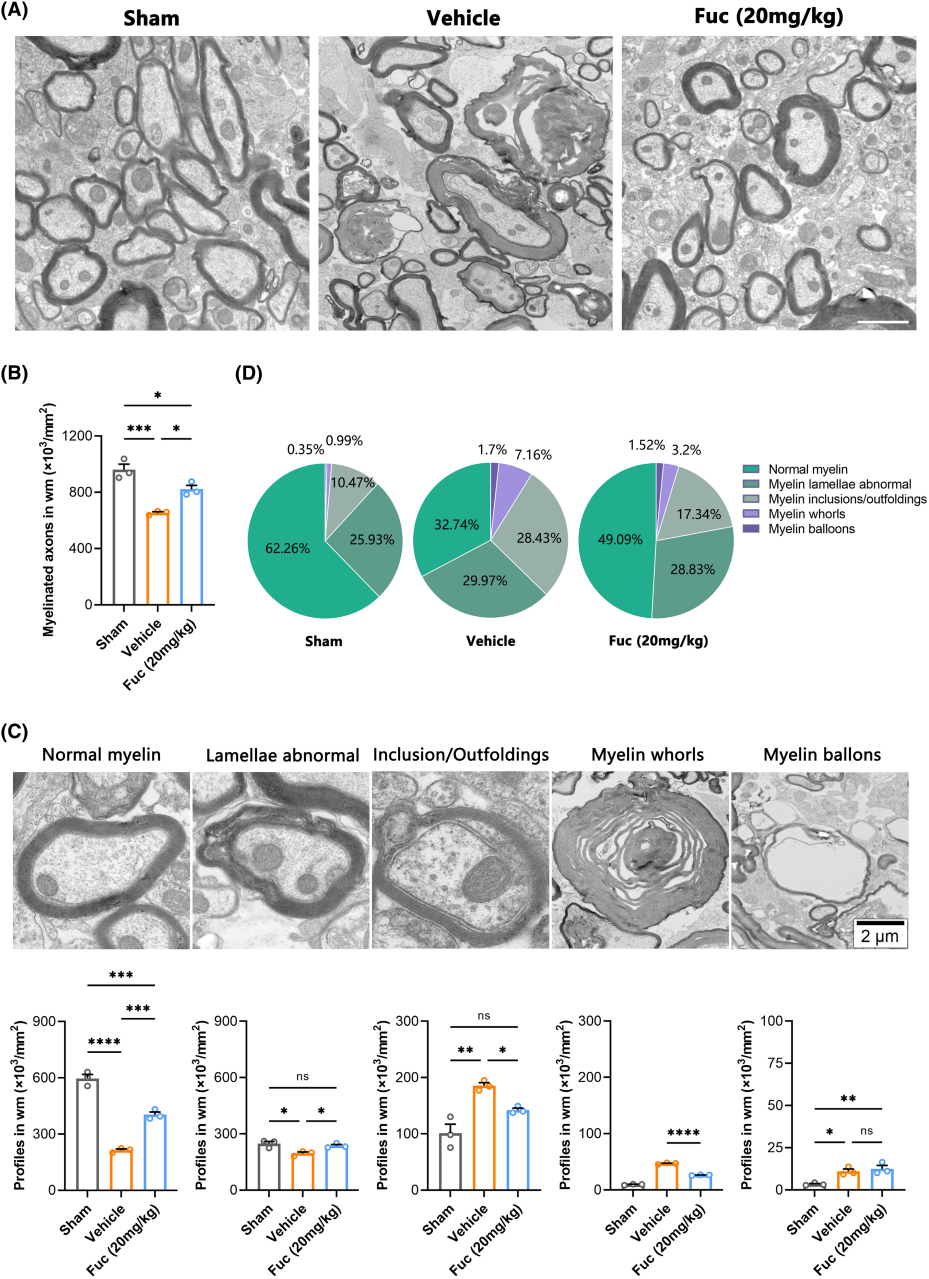
Fucoidan administration promotes myelination of axons and ameliorates myelin pathology in SCI mice. (A) Representative TEM of the ultrastructure of myelin sheaths from SCI mice with different treatments at 28 dpi. (scale bar, 2 μm). Sham, sham‐operated group. Vehicle, SCI group. Fuc (20 mg/kg), SCI treated with 20 mg/kg Fucoidan group. (B) Quantification of the percentage of myelinated axons among total axons within the lesions at 28 dpi. (one‐way ANOVA). (C) Representative TEM images of pathological graded myelin ultrastructural changes and quantitative analysis of the corresponding pathological grades in C, (one‐way ANOVA), see also in Data S1. (D) Pathological‐grade distribution of myelin in each group. The data in B, C passed the Shapiro–Wilk test and exhibit a Gaussian distribution. Data are presented as mean ± SEM, (*n* = 3 mice), *p < 0.05, **p < 0.01, ***p < 0.001, ^****^ *p* < 0.0001, ns, not significant.

To accurately evaluate the remyelination efficiency, we performed TEM to distinguish unambiguously the normal myelin and pathological myelin within the lesion region. Considering the published studies^34^, ^35^ and our own experimental observations, in this work, we established five pathological grades of the myelin sheath ultrastructure in the lesion region (Figure 6C) (Data S1). Compared to the sham group, both the SCI + Veh and SCI + Fuc (20 mg/kg) groups showed varying degrees of demyelination. However, Fuc treatment resulted in extensive remyelination, as evidenced by improved pathological grade of myelin and by the emergence of abundant newly formed thin myelin sheaths (Figure 6A). Subsequently, TEM analysis of all groups revealed a higher proportion of myelinated axons in the SCI + Fuc group (Figure 6B). Notably, compared to the SCI + Veh group, the quantification of myelin pathology level uncovered a higher proportion of normal myelin/abnormal lamellae and a lower proportion of inclusions/outfoldings/myelin whorls in the lesions of the SCI + Fuc group at 28 dpi (Figure 6C,D). Taken together, these results demonstrate that Fuc treatment enhances the number of axons in the injury area and strengthens the myelin sheath structure of axons.

### 3.7 Fuc promotes OPCs differentiation in vitro without affecting their proliferation and apoptosis

**FIGURE 7:**
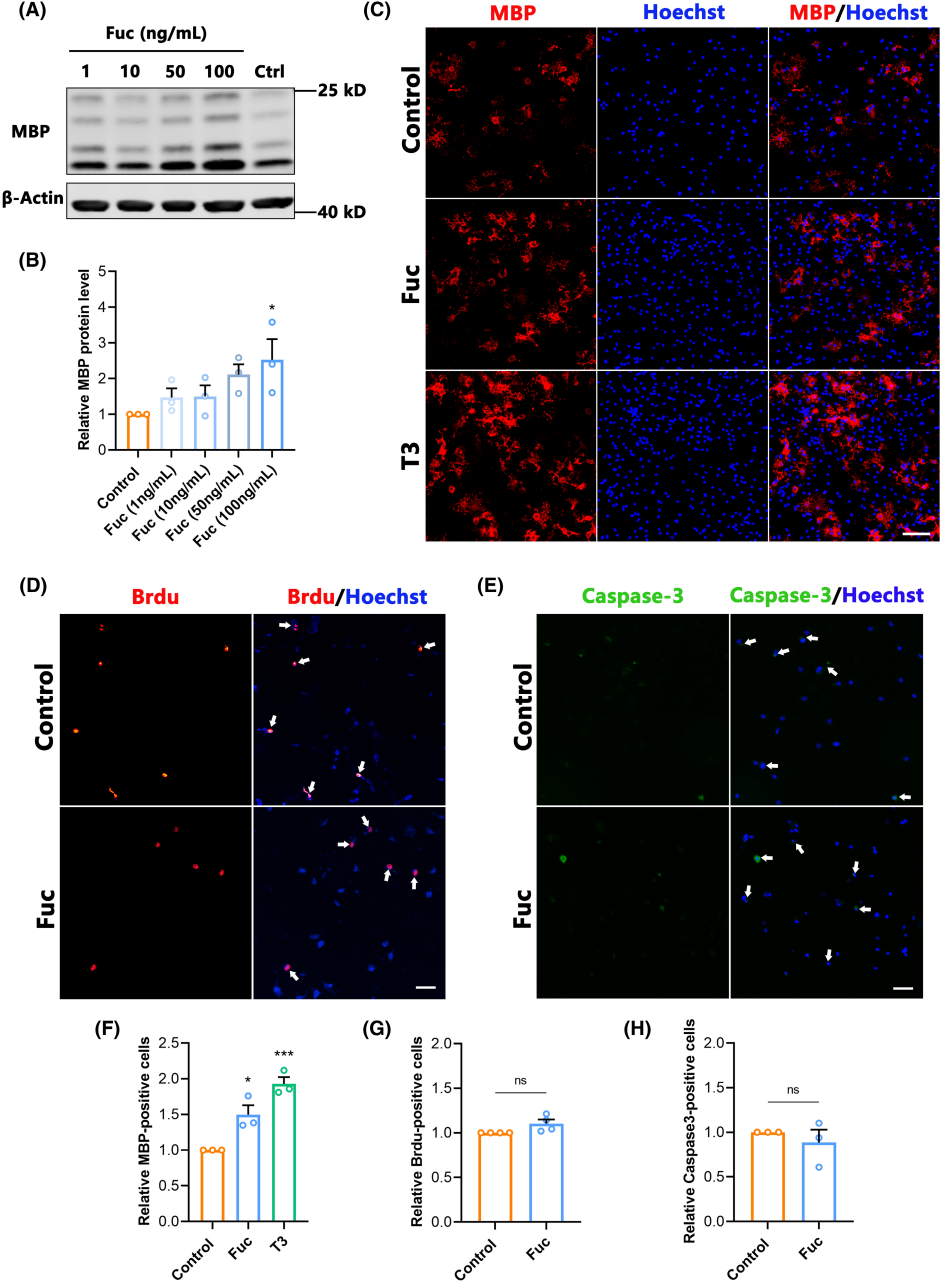
The effect of fucoidan on OPCs in vitro. (A) Western blotting analysis of MBP expression in OPCs under differentiation conditions. (B) Quantification of the relative expression levels of MBP protein as in A. (one‐way ANOVA). (C) Representative images showing MBP^+^ cells (red) in different treatment groups. Nuclei were counterstained with Hoechst (blue). (scale bar, 100 μm). Fuc, cultures with 100 ng/mL fucoidan. T3, cultures with 10 nmol/L thyroid‐hormone 3. (D) Representative images showing BrdU^+^ (red) cells in different treatment groups. Nuclei were counterstained with Hoechst (blue). White arrows mark BrdU^+^ Hoechst^+^ cells (scale bar, 500 μm). Fuc, cultures with 100 ng/mL fucoidan. (E) Representative images showing Caspase3^+^ (green) cells in different treatment groups. Nuclei were counterstained with Hoechst (blue). White arrows mark Caspase3^+^ Hoechst^+^ cells (scale bar, 500 μm). Fuc, cultures with 100 ng/mL fucoidan. (F) Quantitative analysis of the relative percentages of MBP^+^ cells in different treatment groups (one‐way ANOVA). (G) Quantitative analysis of BrdU^+^ cells in the indicated conditions (unpaired two‐tailed Student's). (H) Quantitative analysis of Caspase3^+^ cells in the indicated conditions (unpaired two‐tailed Student's). The data in B, F–H passed the Shapiro–Wilk test and exhibit a Gaussian distribution. Data are presented as mean ± SEM, (*n* = 3–4 wells). *p < 0.05, ***p < 0.001, ns, not significant.

To further reveal the effect of Fuc on OPCs differentiation, we treated OPCs cultured in vitro with different concentrations of Fuc (1, 10, 50, and 100 ng/mL) and normal saline (control) for 72 h. Using MBP as an indicator of differentiated OLs, we found that MBP was upregulated in a dose‐dependent manner after Fuc (50 and 100 ng/mL) administration. Compared to the control group, the MBP expression level and MBP^+^ cells were significantly increased in the 100 ng/mL Fuc treatment group (Figure 7A–C,F), indicating that Fuc indeed promoted the differentiation of OPCs in vitro. Due to the highest expression level of MBP being observed in the 100 ng/mL Fuc treatment group, we use 100 ng/mL as the standard concentration for all in vitro experiments unless otherwise specified. After 3 days of Fuc treatment, the proportion of mature OLs with MBP positivity was significantly higher than that of the control group, which was consistent with the results obtained from thyroid hormone 3, 5, 3′‐triiodo‐l‐thyronine (T3) administration as a positive control (Figure 7C,F).

As the proliferation and apoptosis of OPCs have significant influences on differentiation, we further investigated the effects of Fuc on the proliferation and apoptosis of OPCs. BrdU was used to label proliferating OPCs, and cleaved caspase 3 was used to characterize apoptotic cells. The results showed no difference in the ratio of BrdU^+^ cells between the Fuc treatment and control groups (Figure 7D,G), indicating that Fuc had no effect on OPCs proliferation. In addition, there were no significant differences in the proportion of caspase 3‐positive cells in total cells among Fuc treatment groups compared with the control (Figure 7E,H), indicating that Fuc did not affect the proliferation or apoptosis of OPCs.

### 3.8 Fuc promotes OPCs differentiation by activating the mTOR signaling pathway

**FIGURE 8:**
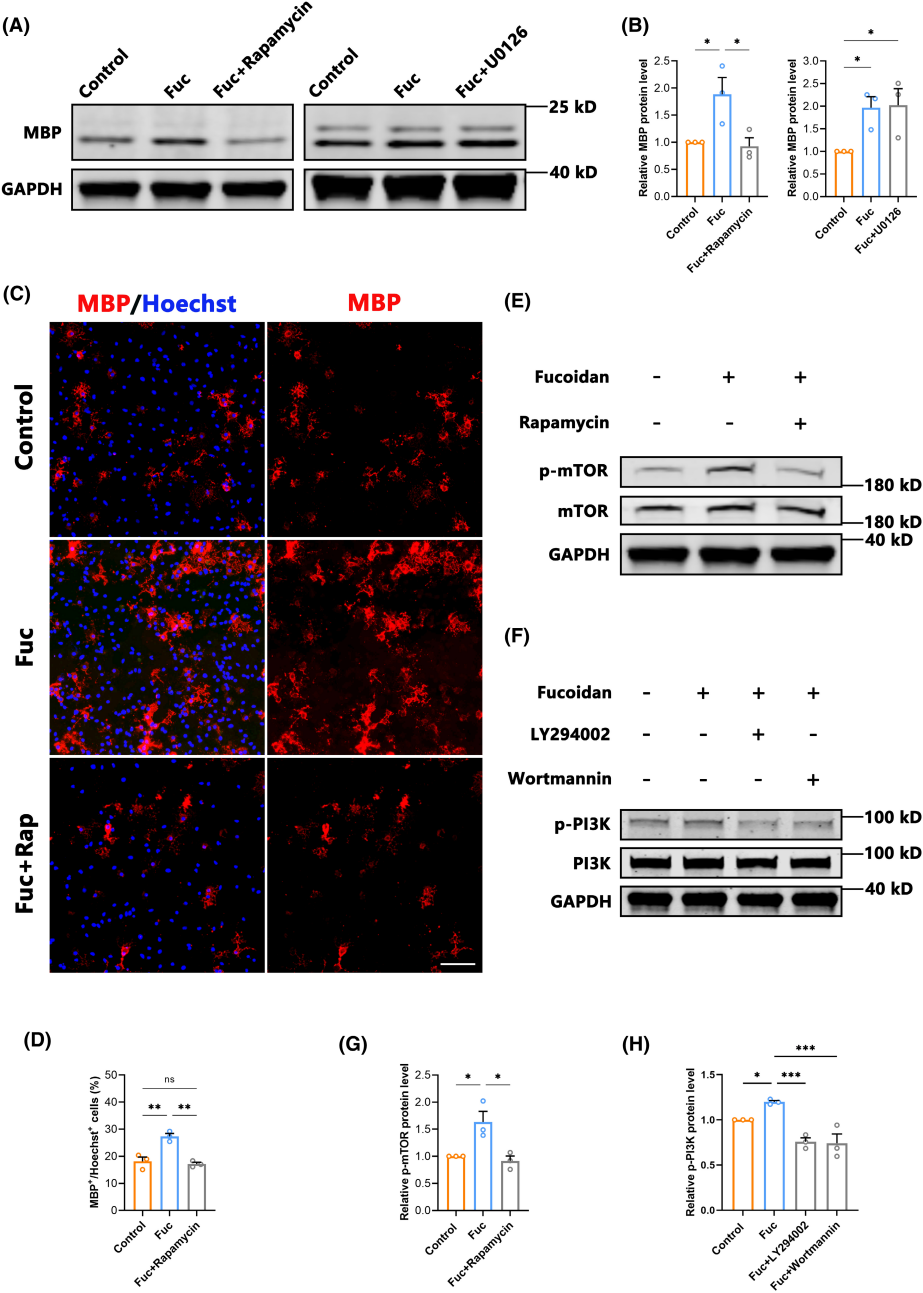
Fucoidan promotes OPCs differentiation through activating the mTOR pathway. (A) Western blotting analysis of MBP expression in OPCs under differentiation conditions. Fuc, cultures with 100 ng/mL fucoidan; Fuc + Rap, cultures with 100 ng/mL fucoidan and 25 nM rapamycin; Fuc + U0126, cultures with 100 ng/mL fucoidan and 4 nM U0126. (B) Quantification of the relative expression levels of MBP protein as in A. (one‐way ANOVA). (C) Representative images showing MBP^+^ cells (red) in different treatment groups. Nuclei were counterstained with Hoechst (blue). (scale bar, 100 μm). Fuc, cultures with 100 ng/mL fucoidan; Fuc + Rap, cultures with 100 ng/mL fucoidan and 25 nM rapamycin. (D) Quantitative analysis of the relative percentages of MBP^+^ cells in different treatment groups. (one‐way ANOVA). (E) Western blots showing the p‐mTOR and mTOR levels in the Fuc + Rap group versus its controls. (F) Western blots showing the p‐PI3K and PI3K levels in Fuc plus Wortmannin or LY294002 group versus its controls. (G) Quantification of the relative expression levels of p‐mTOR protein as in E. (one‐way ANOVA). (H) Quantification of the relative expression levels of p‐PI3K protein as in F. (one‐way ANOVA). The data in B, D, G, and H passed the Shapiro–Wilk test and exhibit a Gaussian distribution. Data are presented as mean ± SEM, (*n* = 3 wells) *p < 0.05, **p < 0.01, ns, not significant.

Previous studies have shown that both the PI3K/AKT/mTOR and Ras/Raf/Mek/Erk pathways play important roles in the development of the OL lineage.^36^, ^37^, ^38^ In order to reveal the pathways targeted by Fuc, we assessed the MBP expression in OPCs cultured in vitro and treated with Fuc plus different inhibitors. Western blotting analysis showed the mTOR inhibitor rapamycin notably blocked the increase of MBP expression in the 100 ng/mL group, whereas the MEK inhibitor U0126 had no blocking effect (Figure 8A,B). Furthermore, the increased MBP^+^/Hoechst^+^ cells with Fuc treatment were diminished after rapamycin was added (Figure 8C,D). In addition, Western blotting analysis showed that the addition of Fuc alone resulted in increased levels of p‐mTOR, whereas the mTOR inhibitor rapamycin inhibited the pro‐differentiation effect of Fuc (Figure 8C–E,G). To further confirm that the differentiation‐promoting effect of Fuc is mediated by the PI3K/AKT/mTOR pathway, we co‐incubated OPCs with Wortmannin and LY294002, which are specific inhibitors for PI3K, an upstream molecule in the AKT/mTOR pathway (Figure 8F), Western blotting analysis showed that both inhibitors of PI3K reduced the phosphorylation level of mTOR to the control level (Figure 8H). These results indicate that Fuc modulates mTOR phosphorylation levels through the PI3K/AKT/mTOR pathway, thereby promoting the differentiation of OPCs.

## 4 DISCUSSION

SCI remains a significant challenge due to its complex pathological basis, primarily characterized by inflammation cascades and the establishment of inhibitory spinal cord microenvironments.^22^ The inflammatory response triggered by SCI involves the activation of immune cells and the release of pro‐inflammatory mediators, which not only exacerbates tissue damage but also disrupts the normal functioning of neurons. The activation of astrocytes has dual effects; while it protects neurons, its excessive activation leads to the formation of glial scars, which in turn creates an inhibitory microenvironment that hinders spinal cord repair.^39^ Additionally, SCI induces widespread apoptosis of neurons, further compromising the structural and functional integrity of the spinal cord.^40^ Moreover, the disruption of myelin sheaths, essential for nerve impulse conduction, is a hallmark of SCI.^41^ Despite the potential for remyelination post‐SCI, its efficacy and pace lag significantly behind the extent of neuronal damage. This intricate interplay of pathological processes poses formidable hurdles for effective SCI treatment strategies. In this study, we demonstrated that the innovative intervention by Fuc mitigates the detrimental effects of SCI and improves functional recovery in mice. In the SCI model, Fuc inhibits inflammation and reverses inflammation polarization, reduces the formation of reactive astrocyte scars, alleviates apoptosis, and promotes remyelination processes.

Following SCI, inflammation is an important defense mechanism against harmful stimuli, but the inflammatory cascade and the sustained and exacerbated inflammation reaction exacerbate nerve damage.^42^ Seeking effective interventions for the inflammatory cascade has been a focus of SCI research.^43^ Fuc, a sulfated polysaccharide rich in L‐fucose, is commonly found in brown algae and sea cucumbers.^44^ It is a non‐toxic polysaccharide with diverse biological functions.^13^ Multiple studies have confirmed the broad anti‐inflammatory effects of fucoidan,^45^, ^46^, ^47^ and in recent years, its neuroprotective effects have been demonstrated.^18^, ^19^ After SCI, resident microglia are rapidly activated, whereas peripheral macrophages infiltrate the injury site several hours later in response to inflammatory chemokines, together constituting the main inflammatory cells involved in the inflammatory response. Microglia and macrophages are classified into classical M1 and M2 types based on the differences in the inflammatory factors they secrete, with M1 typically exhibiting pro‐inflammatory characteristics and M2 exhibiting anti‐inflammatory characteristics, leading to different inflammatory outcomes.^48^ This study confirms that during the first week after injury, which is when the commonly recognized peak of inflammation occurs, Fuc significantly restricts the spread of inflammation and reverses the polarization of inflammation. According to previous studies, this anti‐inflammatory effect is likely to improve neuron survival, alleviate apoptosis, and improve the post‐injury microenvironment.

Apoptosis is a significant mode of cell death for neurons in SCI. The survival of neurons is particularly fundamental for the repair process post‐SCI, and suppressing neuronal apoptosis holds critical significance for SCI treatment.^49^ Apoptosis is regulated by multiple factors, including primary injury, inflammation, oxidative stress, and levels of apoptosis‐related proteins.^50^ Our findings confirm a significant increase in the number of surviving neurons in the injured area following Fuc application, coupled with a notable reduction in neuronal apoptosis. Therefore, we posit that Fuc not only inhibits inflammation but also alleviates neuronal apoptosis, ultimately leading to a remarkable increase in the number of neurons, thus providing greater potential for subsequent neural function restoration.

After SCI occurs, astrocytes promptly undergo activation, contributing to glial scar development at the injury site as part of the reparative process.^39^ Activated astrocytes release a variety of cytokines, including inflammatory mediators and growth factors, which are pivotal in the healing and regenerative mechanisms following injury.^51^ Excessive astrocyte activation can lead to scar formation and the establishment of an inhibitory microenvironment, impeding neuronal regeneration and repair.^52^ Hence, modulation of astrocyte biology represents a promising avenue for the treatment of SCI. Our results demonstrate that Fuc exhibits a dose‐dependent effect in inhibiting astrocyte activation and reducing glial scar formation while also enhancing the density of axons at the injury site, thus providing a favorable foundation for functional recovery.

The recovery of motor function post‐SCI relies significantly on the regeneration of myelin sheaths, provided that axonal regeneration efficiency is moderately ensured. Typically, myelin regeneration hinges on the differentiation and maturation of OPCs.^53^ Following demyelination, OPCs swiftly transition from a quiescent state to an activated state, subsequently undergoing recruitment, proliferation, and differentiation into mature OLs. These mature OLs then generate new myelin sheaths, safeguarding axons and restoring signaling integrity.^54^, ^55^

In our in vivo experiments, immunofluorescence staining revealed an increased population of SOX10^+^ cells within the lesion site after Fuc treatment compared to the control group, indicating that Fuc intervention promoted the survival of OLs lineage. Here, we speculate that this effect may be associated with the anti‐inflammatory and anti‐apoptotic actions of Fuc. Concurrently, under the intervention of Fuc at two different doses, the quantity of CC1^+^ cells increased. Moreover, comparing the two treatment groups, the 20 mg/kg Fuc group exhibited a higher proportion of CC1^+^ cells among SOX10^+^ cells. This facilitation of OPCs differentiation was further visualized through the increased number of MBP‐positive axons, and TEM analysis. These collective findings confirm that intervention with Fuc can enhance the survival of OPCs post‐SCI and promote their differentiation and maturation. Existing evidence establishes the anti‐inflammatory, antioxidant, and neuroprotective effects of Fuc in neurological diseases.^16^ However, its role in promoting OPCs differentiation remains unexplored. Therefore, our studies focused on investigating the impact of Fuc on OPCs differentiation through in vitro experiments.

To further investigate whether Fuc has a differentiation‐promoting effect on OPCs, we conducted additional in vitro experiments. The experimental findings validated that Fuc treatment notably enhanced OPCs differentiation without impacting their proliferation and apoptosis. Eventually, this promotion of differentiation is anticipated to facilitate effective myelin regeneration.

The Ras/Raf/Mek/Erk pathway has been reported to play an important role in the progression of the OLs spectrum.^38^, ^56^ However, our results indicated that an inhibitor of MEK upstream of ERK (U0126) did not block the Fuc‐induced differentiation‐promoting effects of OPCs, suggesting that these effects are independent of the ERK1/2 pathway.

Previous reports indicated that Fuc regulates metabolism through the PI3K/AKT/mTOR axis.^57^, ^58^ AKT, a serine/threonine kinase in the PI3K/AKT signaling pathway, plays a crucial role in regulating cell survival, proliferation, angiogenesis, and metabolism.^59^ Phosphorylated AKT (p‐AKT) directly activates the mammalian target of rapamycin complex 1 (mTORC1) by phosphorylating the Ser2448 site of mTORC1.^60^ mTOR is a downstream effector of AKT signaling, which is quite critical for OPCs differentiation and myelin formation.^61^ Rapamycin is an mTOR inhibitor that inhibits the differentiation of OPCs and the expression of most myelin proteins.^38^ Our experimental results show that Fuc increases the phosphorylation level of mTOR, which can be blocked by rapamycin. Thus, Fuc‐induced OPCs differentiation may be mediated primarily through the PI3K/AKT/mTOR pathway.

Activation of mTOR signals may also regulate the pathology of SCI via anti‐inflammatory and anti‐apoptotic effects. It is reported that fucoidan can alleviate H_2_O_2_‐induced neuronal apoptosis and exert neuroprotective effects by activating the PI3K/AKT pathway,^62^ while medications that promote the AKT/mTOR signaling can alleviate neuronal apoptosis induced by vascular dementia^63^ or traumatic brain injury.^64^ In addition, blocking the activation of the PI3K/AKT/mTOR signaling pathway with PI3K inhibitor not only exacerbates cell apoptosis, but also increases the expression of neuroinflammatory factors TNF‐α and IL‐1β,^65^ while mTOR inhibitor can promote the activation of NF‐κB signaling and increase the production of pro‐inflammatory factors such as IL‐1β and IL‐6 in human monocytes.^66^ These results indicate that fucoidan may play an important role in SCI by activating the PI3K/AKT/mTOR pathway, reducing inflammatory response and neuronal apoptosis. However, due to the complexity of PI3K/AKT/mTOR pathway, its exact role in SCI still needs further investigation.

Based on this experimental study, we hypothesized that Fuc would be an effective drug for the treatment of SCI because it could potentially inhibit inflammation, reduce neuronal apoptosis, improve the regeneration of spinal cord‐injured axons, and promote their myelination. Moreover, we found for the first time that Fuc specifically promotes the differentiation and maturation of OPCs through the PI3K/AKT/mTOR signaling pathway. The findings provide a potential strategy for SCI treatment.

Although the effects of Fuc on neuroinflammation, oxidative stress and neuronal survival in several neurological diseases have been documented, its unique role in SCI remains unexplored. Thus, more detailed studies are needed to enhance our understanding on the roles and mechanisms of Fuc in SCI. In addition, it is crucial of continued in‐depth research on metabolism and distribution characteristics of Fuc in SCI for determining the appropriate therapeutic dosage and duration.

## CONFLICT OF INTEREST STATEMENT

The authors declare no competing financial interests.

## Supporting information


Figures S1–S2

Data S1

Data S2

## Data Availability

The data that support the findings of this study are available from the corresponding author upon reasonable request.
