# Supplementary material for: Fucoidan improving spinal cord injury recovery: Modulating microenvironment and promoting remyelination
Source: CNS Neurosci Ther. 2024 Aug 14;30(8):e14903. doi: 10.1111/cns.14903 (PMC11322593; doi:10.1111/cns.14903)
Supplement: Supplementary file 1 — Figures S1–S2 [file CNS-30-e14903-s002.zip › Data2.docx]

**Supplementary Fig. 1** Fucoidan treatment promotes the survival of cultured neurons in vitro.

1. Representative images showing dead cells (red) and live cells (green) in different treatment groups. (scale bar, 100μm). Sham, no H_2_O_2_ treatment group. Vehicle, H_2_O_2_ treatment. Fuc (50ng/mL), treated with H_2_O_2_ and 50ng/mL Fuc group. Fuc (100ng/mL), treated with H_2_O_2_ and 100ng/mL Fuc group.
2. The ratio of dead cells /total cells in different treatment groups. (one-way ANOVA).
3. Quantification of live cells in different treatment groups (one-way ANOVA).

The data in B, C passed the Shapiro-Wilk test and exhibit a Gaussian distribution. Data are presented as mean ± S.E.M., n=3 wells per group. ^*^ P < 0.05, ^**^ P < 0.01.

**Supplementary Fig. 2** Fucoidan treatment alleviates the inflammatory response of cultured microglia in vitro.

1. Concentration of TGF-α in microglia supernatant of each group (one-way ANOVA).
2. Concentration of IL-10 in microglia supernatant of each group (one-way ANOVA).

The data in A, B passed the Shapiro-Wilk test and exhibit a Gaussian distribution. Data are presented as mean ± S.E.M., (n=3 wells), ^*^ P < 0.05, ^**^ P < 0.01, ^***^ P < 0.01. Vehicle, cultures with LPS. Fuc (50ng/mL), cultures with LPS plus 50ng/mL fucoidan. Fuc (100ng/mL), cultures with LPS plus 100ng/mL fucoidan.
