# Supplementary material for: Fucoidan improving spinal cord injury recovery: Modulating microenvironment and promoting remyelination
Source: CNS Neurosci Ther. 2024 Aug 14;30(8):e14903. doi: 10.1111/cns.14903 (PMC11322593; doi:10.1111/cns.14903)
Supplement: Supplementary file 3 — Data S2 [file CNS-30-e14903-s001.pdf]

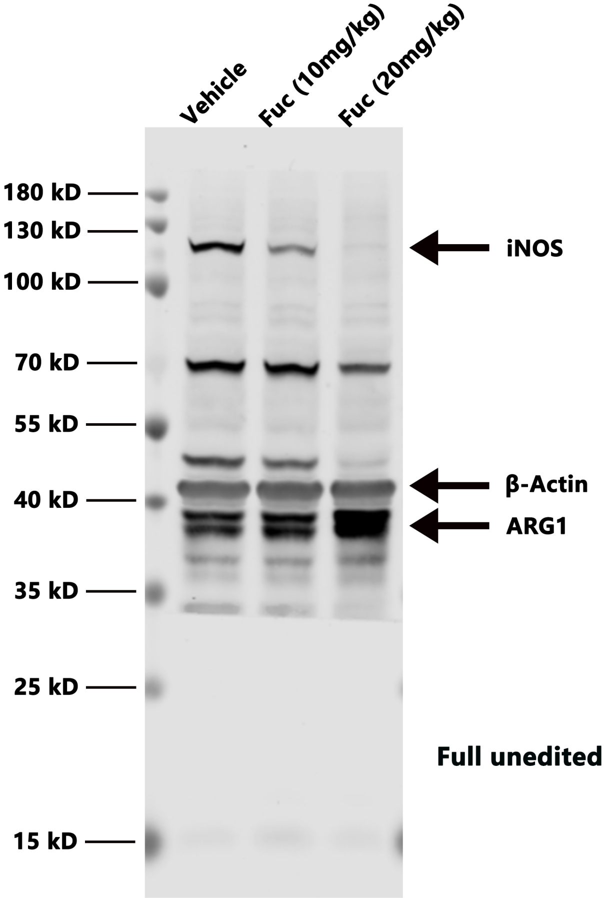

Full unedited blot for Figure 2B

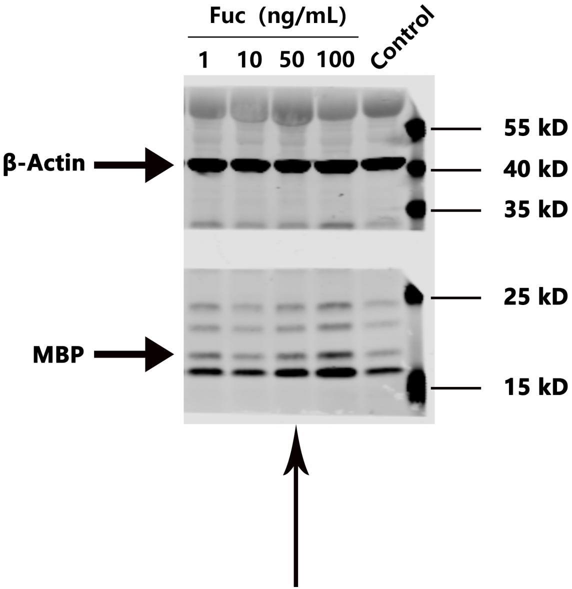

Full unedited blot for Figure 7A

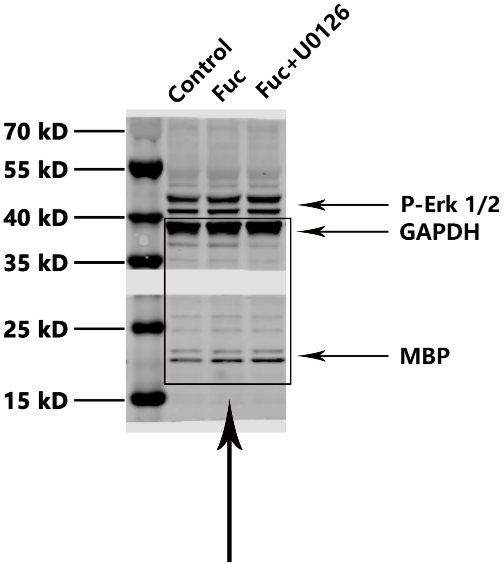

Full unedited blot for Figure 8A

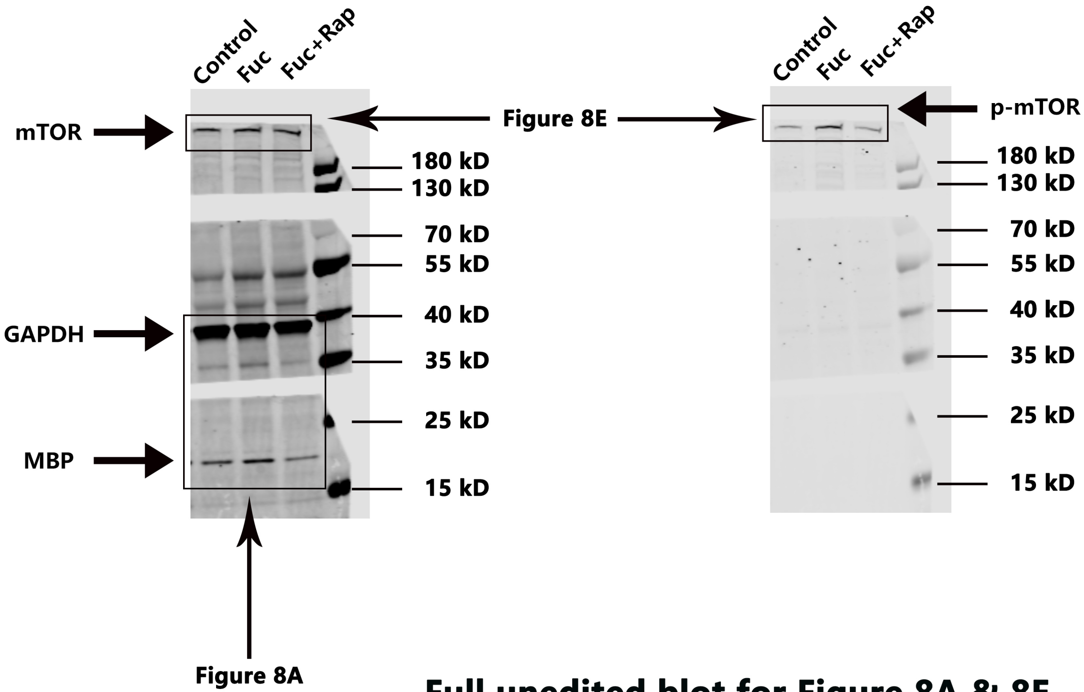

Full unedited blot for Figure 8A & 8E

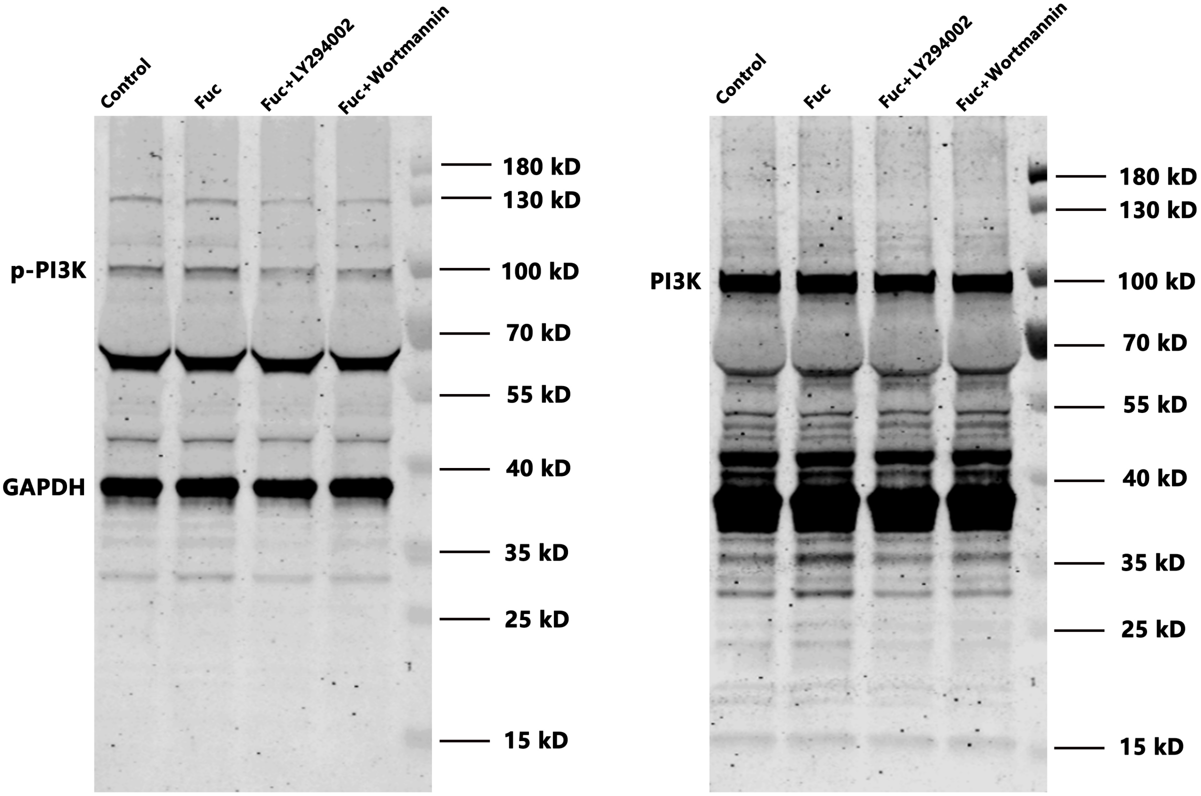

Full unedited blot for Figure 8F
